# Supplementary material for: Preparation and Characterization of Hydroxylated Recombinant Collagen by Incorporating Proline and Hydroxyproline in Proline-Deficient Escherichia coli
Source: Bioengineering (Basel). 2024 Sep 27;11(10):975. doi: 10.3390/bioengineering11100975 (PMC11504287; doi:10.3390/bioengineering11100975)
Supplement: Supplementary file 1 [file bioengineering-11-00975-s001.zip › bioengineering-3183892-supplementary.pdf]

bioengineering

MDPI

*Article*

# Preparation and Characterization of Hydroxylated Recombinant Collagen by Incorporating Proline and Hydroxyproline in Proline-Deficient *Escherichia coli*

**Zhimin Cheng [1], Bin Hong [1], Yanmei Li [1] and Jufang Wang [1,2,*]**

[1] School of Biology and Biological Engineering, South China University of Technology, Guangzhou 510006, China; 202120149891@mail.scut.edu.cn (Z.C.); 202010108485@mail.scut.edu.cn (B.H.); 202010108442@mail.scut.edu.cn (Y.L.)

[2] Guangdong Provincial Key Laboratory of Fermentation and Enzyme Engineering, South China University of Technology, Guangzhou 510006, China

* Correspondence: jufwang@scut.edu.cn

**Table S1.** Strains and plasmids used in this study.

| Strains/plasmids | Genotype or relevant | Source/Ref |
|---|---|---|
| **Strain** | | |
| *E. coli* DH5α | Plasmid cloning | Vazyme Biotech Co., Ltd (Nan Jing) |
| *E. coli* BL21 (DE3) | Protein expression | Vazyme Biotech Co., Ltd (Nan Jing) |
| *E. coli* MG1655 | Protein expression | Vazyme Biotech Co., Ltd (Nan Jing) |
| **Plasmid** | | |
| pET28a | T7 promotor | |
| pCas9 | Cas9 | |
| pTargetF | synthetic DNA construct | |
| pBAD33 | AraC promotor | |
| pET28a-rhCol | rhCol; pBR322 ori; | This study |
| pTargetF-*proC* | sgRNA-*proC* | This study |
| pTargetF-*ompT* | sgRNA-*ompT* | This study |
| pTargetF-*lon* | sgRNA-*lon* | This study |
| pBAD33-T7RNAP | T7RNAP | This study |

**Table S2.** Primers used in this study.

| primer | sequences |
|---|---|
| KZ-T7RNAP-F | cggggatcctctagagtcgacATGAACACGATTAACATCGCTA |
| KZ-T7RNAP-R | atccgccaaaacagccaagcttTTACGCGAACGCGAAGTC |
| sgRNA-F | cctaggtataatactagtAATTTATACGCCTGGGCGCGgttttagagctagaaatagc |
| sgRNA-R | ACTAGTATTATACCTAGGACTGAGC |
| proC-up-F | agggtaccatatgggaattcGCTATCTGGTCCGCCG |
| proC-up-R | TGCCTCACTCCTGCCGTGAA |
| proC-dn-F | TTCACGGCAGGAGTGAGGCATGACTTTCGCCGGACGTCAG |
| proC-dn-R | ccgccaaaacagccaagcttCCGCGACGGCATGACG |
| ompT-sgRNA-F | cctaggtataatactagtCAGTAACCCCGGAACCTGGAgttttagagctagaaatagc |
| ompT-up-F | agggtaccatatgggaattcAACGGATAAGACGGGCATA |
| ompT-up-R | AAAAGTTCTCCATTCAATCGTT |
| ompT-dn-F | CGATTGAATGGAGAACTTTTGAACGCCAACTAAAATTTCC |
| ompT-dn-R | ccgccaaaacagccaagcttGTTTAATAAAAAAAAGATTAAGGGA |
| lon-sgRNA-F | cctaggtataatactagtCATACGGACATATTTACGCCgttttagagctagaaatagc |
| lon-up-F | agggtaccatatgggaattcTTGCAGCCGTGCGTAAG |
| lon-up-R | TGACCTCGCGCAAAATG |
| lon-dn-F | GTGCATTTTGCGCGAGGTCAAGAGCTCTCTCTTAGTTTAATTTCC |
| lon-dn-R | ccgccaaaacagccaagcttGATAAAGTGATTTCCCACCG |
| YZ-pBA-S | gcgttaaacccgccacca |
| YZ-pBA-A | cctttcgttttatttgat |
| proC-up1000-S | GGCGGTCATTTTGCTGTG |
| proC-dn700-A | GCGGGTCTGGTGTTGAT |
| ompT-up700-S | GATTGTCACAGAGTGTCGTATGC |
| ompT-dn700-A | GCTTGTTTTTTCTCTGTTTGAAG |
| lon-up1000-S | GTTTCAGCTCACGTGCGTAC |
| lon-dn1000-A | GGTGAAGACGTTGAAAACATC |
